# Further Empirical Evidence on Patrick Hughes’ Reverspectives: A Pilot Study

**DOI:** 10.3390/vision5010002

**Published:** 2020-12-26

**Authors:** Alessandra Galmonte, Mauro Murgia, Fabrizio Sors, Valter Prpic, Tiziano Agostini

**Affiliations:** 1 Department of Medicine, Surgery and Health Sciences, University of Trieste, 34149 Trieste, Italy; fsors@units.it; 2 Department of Life Sciences, University of Trieste, 34128 Trieste, Italy; mmurgia@units.it (M.M.); agostini@units.it (T.A.); 3 Faculty of Health and Life Sciences, Institute for Psychological Science, De Montfort University, The Gateway, Leicester LE1 9BH, UK; valter.prpic@dmu.ac.uk

**Keywords:** reverspective, texture, spatial arrangement, illumination

## Abstract

Reverspectives are paintings created by the English artist Patrick Hughes. They are 3D structures, for example, pyramids or prisms, which elicit an illusory depth perception that corresponds to the reverse of the physical depth layout. Rogers and Gyani state that “*the perspective information provided by a simple grid of vertical and horizontal lines on a slanting surface can be just as powerful as the information provided by a rich, naturalistic scene*”. The present experiment was aimed to further investigate this perspective reversal. Three independent variables were manipulated: (1) texture components (i.e., vertical, horizontal, and oblique lines components), (2) texture spatial arrangement (i.e., Hughes-type “perspective” grid vs. equidistant “no perspective” grid), and (3) illumination direction (i.e., homogeneous illumination, light from above, and light from below). The dependent variable was the “critical distance”, namely, the distance between an approaching observer and the stimulus at which the illusory depth perception of concavity/convexity switched to the actual perception of convexity/concavity. The results showed that a stronger illusion is elicited by: (a) a Hughes-type texture spatial arrangement; (b) a complete grid texture composition, having both vertical and horizontal, and oblique components; and (c) illumination from below, as opposed to the condition in which light is coming from above.

## 1. Introduction

Starting in 1990, the English artist Patrick Hughes decided to devote himself entirely to one particular type of artwork that he invented, which he named, using a peculiar neologism, reverspective.

Reverspectives are painted on 3D structures, consisting of protruding solid geometric elements shaped as partially overlapping prisms or pyramids, and represent perspective spaces that give the impression of reverse depth. The reverspectives arouse in the observer a very strong illusion: the volume of the protruding solids is phenomenally inverted, creating a completely fictitious new space, in which what is physically closer is perceived as more distant and vice versa. The illusion is enhanced by the observer’s movement: if s/he moves sideways or from top to bottom, the impression of perspective reversal becomes more stable and, when the illusion begins to fail, the movement of the observer may restore it, even if sometimes it fails. The reverspective illusion strength is then also closely connected to the relative movement that the observer performs in front of the artwork: the displacement (in any direction) of the observer acts as a “catalyst” for the illusion.

However, Hughes’ reverspectives do not strictly follow the rules of geometrical perspective, but loosely: the distribution of the objects in depth is somewhat disproportionate, and the vanishing points multiply. The phenomenon is bistable: the observer perceives the illusion until the “switch-off” (i.e., the moment at which a change between the illusory perception of concavity/convexity and actual perception of convexity/concavity) takes place, namely, a sudden perceptual change shows the actual 3D physical structure of the artwork. The reverspectives create a situation of conflict between different depth cues, however, before the switch-off occurs, the pictorial cues that suggest to the observer the reversal of the actual 3D structure clearly prevail; the other cues—such as the motion parallax and the retinal disparity-remain in the background. 

Hughes acknowledges that in his early works the illusion was not produced in a particularly effective way; over the years, he developed his techniques, gradually finding the elements necessary to achieve artworks that elicited a strong illusory effect through specific use of the lines of perspective, of shadows and shading, and of 3D structure [1]. 

Another crucial aspect of the illusion is the fact that Hughes did not adhere strictly to the rules of geometrically correct perspective. 

Therefore, it seems plausible to assume that this effect should be based on the combination of various factors that interact with each other: the perfect illusion is then the result of the correct mix of all these factors. 

Some researchers tried to decouple the reverspective cues in order to understand which is the factor that most determines the 3D illusory phenomenon [1,2,3,4].

Cook et al., [1] created inverse perspectives by varying the number of depth cues present and by differentiating the experimental stimuli on the basis of the foreground objects’ color, of the presence of self/cast shadows, of the presence/absence of a grid of perspective lines, and of the complexity of the represented 3D objects. Papathomas [2] studied three different reverspectives, one completely painted, one with contours only, and one entirely white, while later, Papathomas and Bono [3] investigated whether a 180° rotation of the stimulus led to significant perceptual differences. These researches experimentally demonstrated that the more complex and realistic stimuli are the ones that produced a more lasting and effective illusory phenomenon.

Rogers and Gyani [4] demonstrated that the question is far more complex. The authors systematically manipulated inverse reverspectives textures by all having the same 3D structure but progressively decreasing the details and “true-to-life” components. Rogers and Gyani studied 7 stimuli. Among them, they tested a perspective photograph, (i.e., stimulus #1: the “New College” stimulus) a grid of prospectively modulated black lines, a solid made by contours only, and a cobblestone texture containing size and density gradients, without any straight line. The authors discovered that the latter stimulus, with size and density gradients, although its perspective is correct, is the weakest in evoking the inverse 3D illusory effect; moreover, the perspective photograph stimulus, is slightly less effective, whereas, on the contrary, one of the most effective stimuli is the grid of lines prospectively modulated. More specifically, Rogers & Gyani stated that “*the critical distance [...] was remarkably similar for six out of the seven reverspectives [...]. The average critical distances for the ‘New College’ reverspective (85.5 cm) and the ‘outline’ reverspective (88.5 cm) were slightly larger than for the remaining four (average, 53.6 cm)*”. What Rogers and Gyani results show is that the geometrical component of the reverspective consisting of straight vertical, horizontal and oblique lines converging towards the vanishing points, is the factor that most influences the illusory effect, while texture gradient, realism and familiarity, contrary to Papathomas [2] and Cook [1], seem to play a relatively minor role: it seems that, at least for the stimuli they tested, the ones that were the most realistic and correctly built from the perspective point of view, turned out to be those that elicit less the illusion. Therefore, according to Rogers and Gyani [4], the most important factor in giving rise to reverspective is the presence of lines that converge to the vanishing points.

Deregowski and Parker [5] explore the theory of Kazimierz Bartel (1882–1941), who, starting from a purely geometrical analysis of the perspectives present in artistic works of different ages and geographical locations, investigated it experimentally by asking observers to reproduce perspective geometric structures in different modalities, obtaining results that show how the human visual system is extremely lacking precision: the more the observer is involved in the representation of space, the further he moves away from realistic representation. It is as if, on a phenomenal level, we tend to “rotate” the shortened surfaces by making them approach the fronto-parallel plane. The same type of deformation can be found in the geometric structure underlying the reverspective. 

Bartel’s theory is further corroborated by Deregowski and McGeorge [6] results, which demonstrate that objects are more recognizable if they are reproduced with well-visible contours and on a fronto-parallel plane. The need to recover the shape of typical contours from images that either hide them or show them from atypical viewpoints would lead the mind to make an effort that makes their discrimination more difficult and tiring. 

Looking at the works of several artists (even those who paint with a very realistic style) and at children’s drawings, it could be concluded that the visual system prefers an “incorrect” perspective construction, flattening and shortening the surfaces, that is, rotating the planes in such a way as to bring them closer to the observer’s frontal plane. This rotation occurs at the phenomenal level, and the painters, probably unconsciously, bring it in their paintings, thus making the image simpler because it makes the typical contours of the represented object better identifiable [6]. Therefore, paintings are often quite different from what is prescribed by geometric drawing rules, but, nonetheless, they closely resemble Hughes’ stimuli [7].

According to Deregowski and Parker [8,9,10], it is plausible to presume that oblique lines play a dominant role: if indeed, the oblique lines would play such an important role in depth perception, one might expect that a reverspective consisting exclusively of converging oblique lines could be particularly effective or, at least, more effective than one constituted by vertical and horizontal lines only.

Three main facts can be considered as starting points:

(a) Reverspectives produce an illusory depth perception that does not correspond to the physical structure of the object, as the perceived geometrical structure corresponds to the illusory reversal of the actual 3D structure of the object;
(b) Reverspectives, despite the fact that they give rise to a 3D illusion, from the geometrical structure point of view, have little in common with the correct perspective: the proportion that regulates the reduction of the dimension of the elements distributed in depth is completely arbitrary, and there can be multiple vanishing points;

The present research started from Malisano and Agostini’s [11] paper and is aimed to try to better understand which variables make Hughes’ reverspectives so strong. For this purpose, the original Hughes’ geometric texture is manipulated in terms of both geometrical components and of their spatial arrangement, to identify the most effective variables that determine the observed illusory effects. In this effort, we try to isolate the components of reverspectives, to investigate the relative strength of the factors involved. Moreover, we investigate a factor that was never studied before, that is, the influence of the direction of illumination.

## 2. Experiment and Methods

### 2.1. Overview of the Experiment

The experiment presented in this work can be considered as a logical continuation of the experiments performed by Cook et al. [1], by Papathomas [2,3], and, above all, by Rogers and Gyani [4], seeking a deeper understanding of the role of visual cues. All the above-cited works are aimed at examining the relative strength of various components/cues in the visual stimuli of reverse perspectives, in producing the illusory effect of depth reversal.

The present experiment was aimed to clarify the role played in the production of the reverspective phenomenon by three variables, that is: (1) the geometrical texture components (i.e., vertical, horizontal, and oblique lines), (2) the texture spatial arrangement (i.e., Hughes-type perspective grid vs equidistant grid), and (3) illumination direction (i.e., homogeneous illumination, light from above, and light from below). 

As regards the type of texture, geometrical elements have only been considered in this paper, starting from what Rogers and Gyani [4] employed; in particular, we examined which of the components of the grid texture that is composed of horizontal, vertical, and oblique lines, built by following Hughes’ perspective logic, are the most effective ones. 

The aim was to try to understand how the illusory effect varied by manipulating the following two factors:

● Texture components (i.e., “complete” vs.” incomplete” grid conditions):
o vertical and horizontal lines (squares) plus oblique lines “complete grid” vs. vertical and horizontal lines (squares) only “incomplete grid”
● Texture spatial arrangement (i.e., “perspective” vs. “no perspective” grid conditions):
o Hughes-type “perspective” grid vs. equidistant “no perspective” grid

As regards the illumination direction variable, it must be noted that this is the first time that it is considered in a reverspective experimental study. In all the papers cited above the reverspectives were illuminated by a diffused frontal light. We considered three illumination direction conditions:

o homogeneous illumination (i.e., the stimulus was illuminated with the same intensity from all four sides, namely left, right, top and bottom)
o predominant illumination from above
o predominant illumination from below

To summarize, the purpose of the experiment presented in this work was to investigate different reverspectives characterized by different geometric textures—in terms of both their geometrical components and of their spatial arrangement-derived from the artworks of Patrick Hughes, with different illumination directions, to try to understand whether there are any significant differences in the effectiveness of the illusion, measured by using the “critical distance” index (i.e., the distance where, during the approach of the observer to the stimulus, the switch-off takes place): the smaller the critical distance, the stronger is the illusion.

### 2.2. Methods

#### 2.2.1. Observers

Fifteen undergraduate students were tested, and they received credits for their participation. All were volunteers and naïve as to the purpose of the experiment. Informed consent was obtained for experimentation with human subjects. The study adhered to the tenets of the Code of Ethical Principles for Medical Research Involving Human Subjects of the World Medical Association (Declaration of Helsinki) and was approved by the Trieste University’s Ethics Committee (Project identification code: 101/2019, 4 December 2019). All observers had normal vision: any sight defect, even if corrected to normal, was an exclusion criterion.

#### 2.2.2. Apparatus

The experimental apparatus consisted in a fixed vertical plane on which the bases of the experimental stimuli were placed. This plane was made by a rectangular panel of hardboard (50 cm wide by 44 cm tall). We constructed a frame made of a half-a-centimeter-thick sheet of polystyrene that was painted black; the dimensions of the frame were the same as those of the panel. We cut a rectangular opening at the center of this frame that was 30 cm wide and 24 cm tall (i.e., the same dimensions of the stimuli) and we glued the frame on the plane. The opening provided the base on which the pyramids—made by white cardboard—could be inserted. This apparatus was built by a system of magnets, allowing the experimenter to quickly and easily change the experimental stimulus to be presented to the observer. Moreover, the apparatus height (i.e., its distance from the floor) was adjustable, to allow a regulation of the geometric center of each stimulus in such a way that it was always aligned with the observer’s gaze (i.e., at the same height as the observer’s eyes).

To control the illumination variable, a 1.50 m wide, 2 m high and 3.5 m long tunnel of heavy black fabric, supported by a tubular structure, was built. The lab door and windows were shielded in order to obtain an almost completely dark environment, to further exclude any extraneous light source that could affect the control of the illumination. Moreover, a black cloth was placed (the experimental stimulus stretched from one side of the tunnel to the opposite one) in such a way that the observer could not see the support of the hardboard.

The stimulus illumination was obtained by using four identical lamps placed above, below, at the right and at the left of the experimental stimulus. They were mounted inside the fabric tunnel, wrapped in white tissue paper boxes to make the light more homogeneous and diffused. Moreover, the lamps were also shielded behind a black painted cardboard in such a way that the observer could not see their position inside the tunnel. 

To allow easy and quick measurement of the switch-off distance, a graduated adhesive strip has been placed on the floor from the experimental stimulus position to the starting point of the observer, which was 7 m away from it. The observer’s perceptual outcome of the experimental apparatus was that of a dark tunnel in which, at its end, at the height of her/his gaze, the only thing s/he could see is an illuminated pyramid.

#### 2.2.3. Stimuli

Stimuli were solid convex symmetric 3D pyramids placed pointing toward the observer, and were perceived afar as a tunnel, according to Hughes reverspective. They were made up of white cardboard, on which different types of geometric textures composed by black lines were depicted; the overall background color was black. The square base side measured 30 cm, the pyramid height was 24 cm. Each stimulus subtended a visual angle of 17°4′48′′ at a distance of one meter, and of 2°18′ at a distance of seven meters. The stimuli resembled Patrick Hughes’ reverspectives geometrically, in which the texture has been varied to manipulate the geometric features, i.e., the geometrical components and their spatial arrangement. 

**Figure 1:**
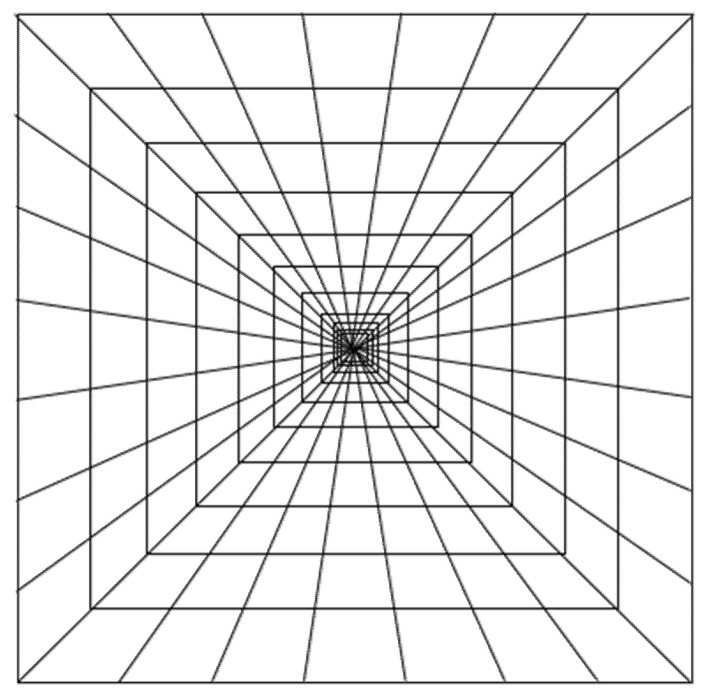
Schematic of experimental stimulus 1 (CP): COMPLETE (obliques + squares) HUGHES-TYPE “PERSPECTIVE” GRID.

**Figure 2:**
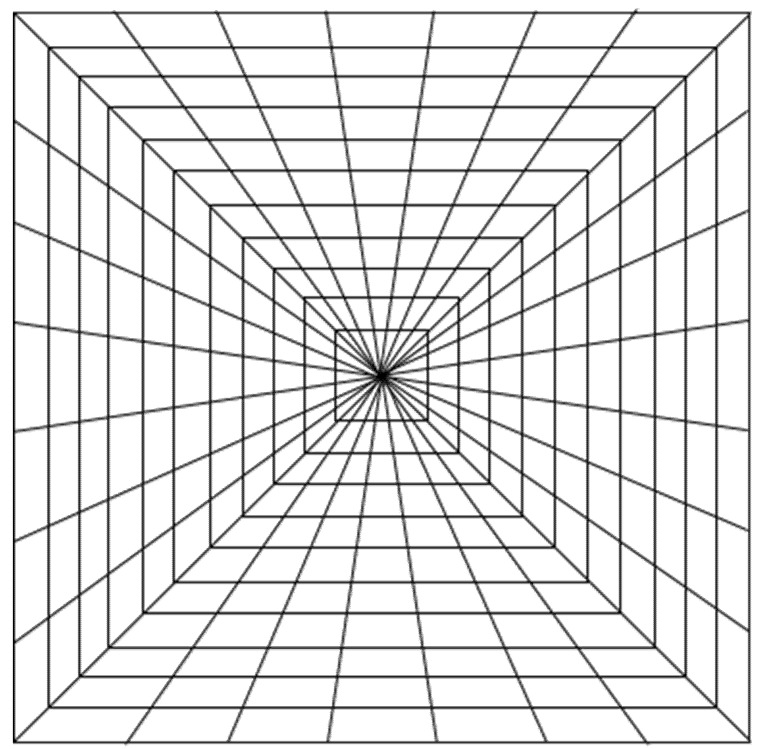
Schematic of experimental stimulus 2 (CN): COMPLETE (obliques + squares) EQUIDISTANT “NO PERSPECTIVE” GRID.

**Figure 3:**
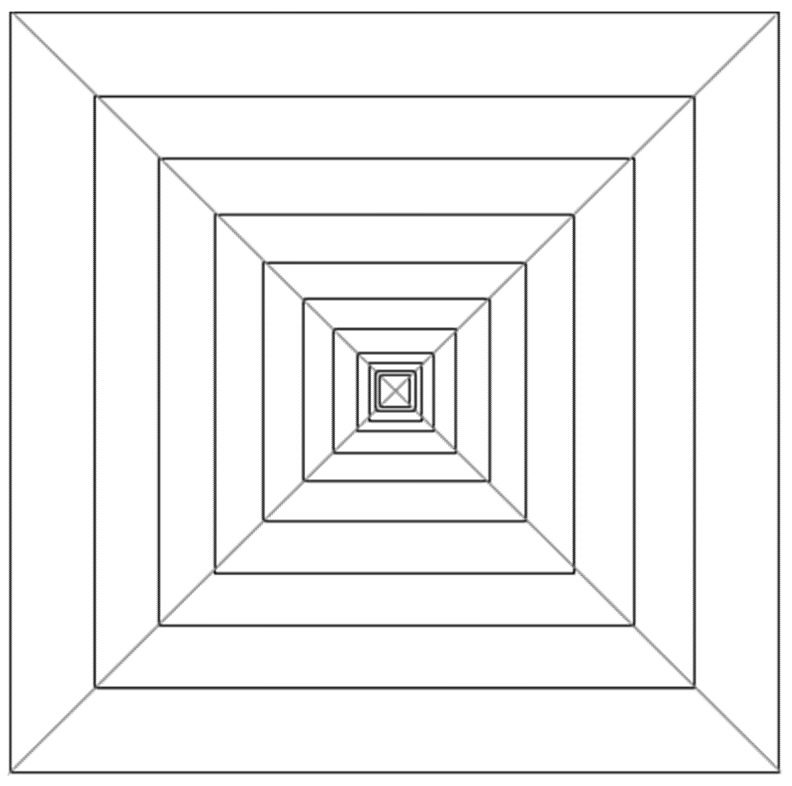
Schematic of experimental stimulus 3 (IP): INCOMPLETE (squares only) HUGHES-TYPE “PERSPECTIVE” GRID.

**Figure 4:**
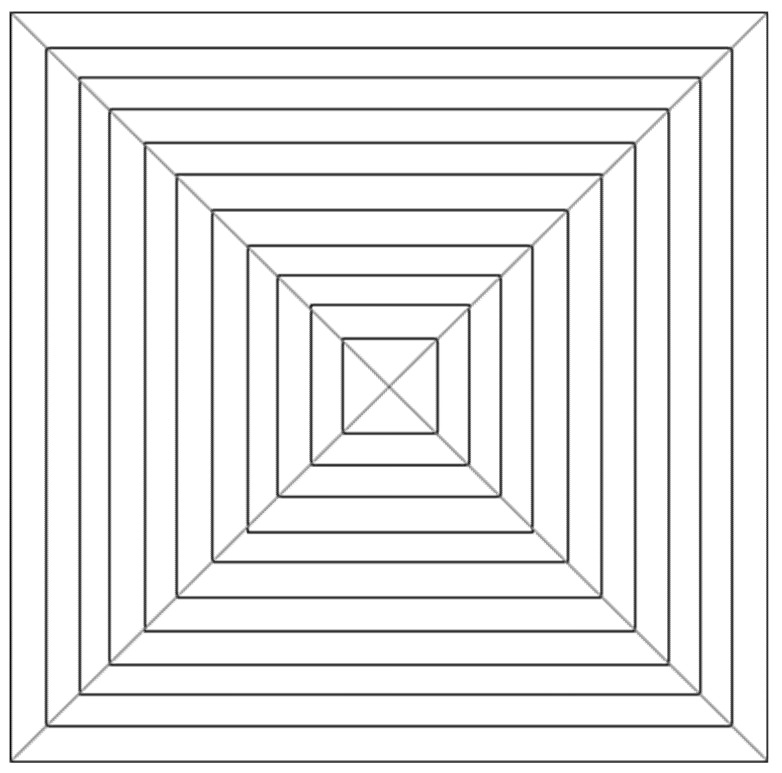
Schematic of experimental stimulus 4 (IN): INCOMPLETE (squares only) EQUIDISTANT “NO PERSPECTIVE” GRID.

The four experimental stimuli (Figure 1, Figure 2, Figure 3 and Figure 4) were the result of the combination of the two levels of the two tested variables, that is: (a) Texture components (i.e., “C: complete” vs. “I: incomplete” grid conditions), and (b) Texture spatial arrangement (i.e., “P: perspective” vs. “N: no perspective” grid conditions):

(1) CP: COMPLETE (obliques + squares) HUGHES-TYPE “PERSPECTIVE” GRID: The pyramid texture was composed of oblique black perspective lines, starting from the pyramid vertex to its base, modulated according to the perspective laws (i.e., very thin near the vertex and gradually becoming thicker), plus vertical and horizontal orthogonal lines (squares) painted by following the same method used by Hughes to design his own reverspective: the distance between the squares was diminishing as we approached the vertex, starting with an initial distance and getting smaller by a ratio of about 1:7 that was chosen arbitrarily (see Figure 1). The width of the lines was also diminishing as they approached the pyramid’s vertex. In addition to the black oblique lines on the four edges of the pyramid, there were six oblique lines that were equidistantly painted on each side of the pyramid.
(2) CN: COMPLETE (obliques + squares) EQUIDISTANT “NO PERSPECTIVE” GRID: The pyramid texture was composed of oblique black perspective lines, starting from the pyramid vertex to its base, modulated according to the perspective laws (i.e., very thin near the vertex and gradually becoming thicker), plus vertical and horizontal orthogonal lines (squares) that were equidistantly spaced, and the lines were all of the same thickness (see Figure 2).
(3) IP: INCOMPLETE (squares only) HUGHES-TYPE “PERSPECTIVE” GRID: the stimulus was identical to that described in 1, but without the oblique lines (see Figure 3).
(4) IN: INCOMPLETE (squares only) EQUIDISTANT “NO PERSPECTIVE” GRID: the stimulus was identical to that described in 2, but without the oblique lines (see Figure 4).

It must be noted that the width of the lines in Figure 1, Figure 2, Figure 3 and Figure 4 is not shown to vary as it did in the actual stimuli.

As regards the three different illumination conditions, the luminance values, measured with a LS 100 photometer (Konica Minolta, Ramsey, NJ, USA, measurement range from 0.001 to 229.900 cd/m^2^) from the distance of one meter from the stimulus, were:

(1) homogeneous illumination (the light reached all the four faces of the pyramid with approximately the same intensity): ≈21.00 cd/m^2^ on all sides.
(2) illumination coming mainly from above: 20.07 cd/m^2^ on the top (illuminated) side, and 0.57 cd/m^2^ (average) on the shaded areas. Even though the three sides of the pyramid that were in shadow were slightly different in terms of luminance, these differences were so small that we decided to report the average luminance only.
(3) illumination coming mainly from below: 20.07 cd/m^2^ on the illuminated area, and 0.57 cd/m^2^ (average) on the shaded areas. Even though the three sides of the pyramid that were in shadow were slightly different in terms of luminance, these differences were so small that we decided to report the average luminance only.

#### 2.2.4. Procedure

Observers were tested individually and viewed the stimuli binocularly. The experiment started by leading the observer at a 7 m distance from the experimental stimulus, and by asking her/him to describe what s/he perceived, to check whether s/he was able to perceive the illusion. The participants who were not able to perform the task (i.e., who did not perceive the illusion), have not been included in the sample. The observer was then asked to gradually approach the experimental stimulus, advancing along the graduated adhesive strip glued to the floor, and to stop when the illusion was lost, that is, at which distance the inversion of the initial illusory 3D percept occurred; in other words, s/he had to indicate when s/he was able to recognize the true 3D geometry of the stimulus. Observers were not allowed to move laterally but only forward and backwards along the linear straight path defined by the graduated strip placed on the floor. Moreover, observers have been asked to walk as slowly as possible to allow a measure most precise as possible of the switch-off distance.

A first (practice) trial with a stimulus different from the experimental ones was performed to check whether the observer understood the task. The practice stimulus was a pyramid built according to correct perspective laws, that is, its texture was composed of oblique black perspective lines, starting from the pyramid vertex to its bases, modulated according to the correct perspective laws, plus vertical and horizontal orthogonal lines (squares) painted by following the correct perspective laws. The width of the lines was also diminishing as they approached the pyramid’s vertex, according to perspective laws. Once sure that the task was clear for the participant, s/he was asked to turn her/his back to the stimulus to allow the experimenter to replace it with the first experimental one. Then, the observer turned back towards the experimental stimulus and the experiment started. This procedure was identical for each of the 4 experimental stimuli. The experimental stimuli order was randomized. Two measures have been taken for each stimulus.

## 3. Results

To preserve the trends across conditions, data have been normalized following the procedure of Papathomas and Bono [3], that is, each observer’s critical-distance has been divided by the mean critical distance for that observer across the conditions. These normalized distances have been then used to obtain averages across observers.

**Figure 5:**
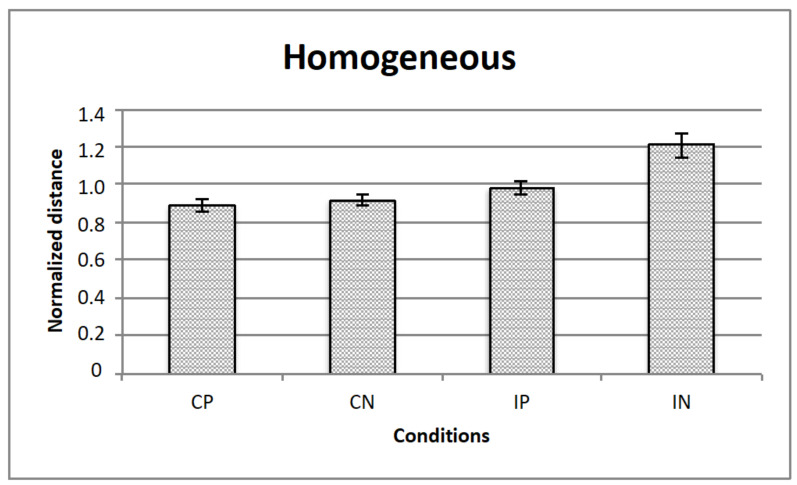
The figure represents the data for homogeneous illumination: The horizontal axis represents the experimental conditions (i.e., CP, CN, IP, IN), while the vertical axis is for the normalized critical distance (i.e., averaged across observers). Standard errors are reported.

**Figure 6:**
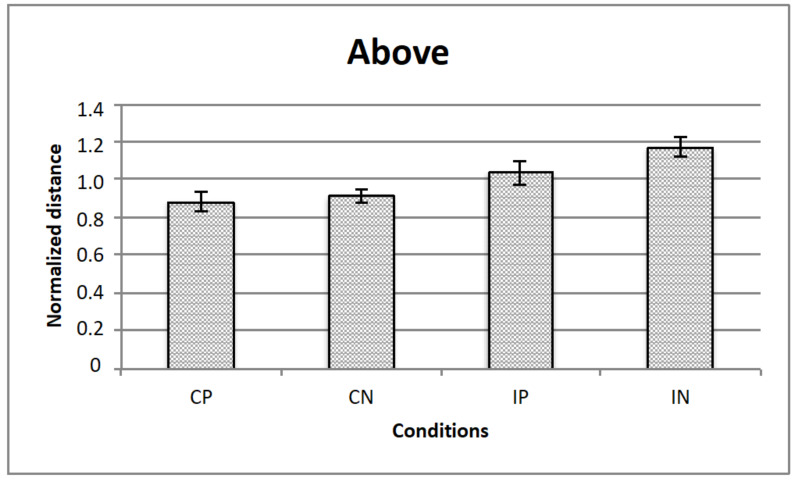
The figure represents the data for the illumination from above: The horizontal axis represents the experimental conditions (i.e., CP, CN, IP, IN), while the vertical axis is for the normalized critical distance (i.e., averaged across observers). Standard errors are reported.

**Figure 7:**
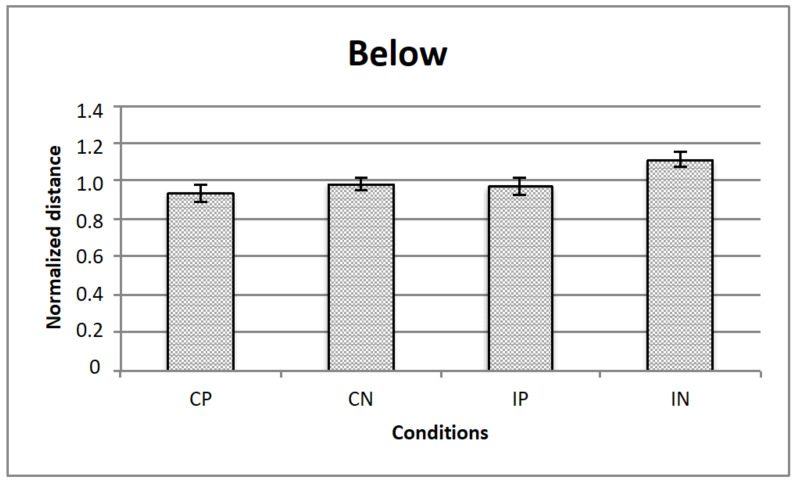
The figure represents the data for the illumination from below: The horizontal axis represents the experimental conditions (i.e., CP, CN, IP, IN), while the vertical axis is for the normalized critical distance (i.e., averaged across observers). Standard errors are reported.

**Figure 8:**
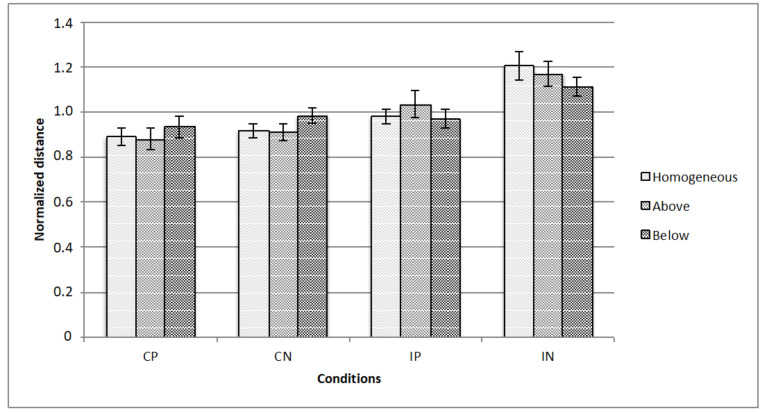
The figure represents the data for all the illumination conditions: The horizontal axis represents the experimental conditions (i.e., CP, CN, IP, IN), while the vertical axis is for the normalized critical distance (i.e., averaged across observers). Standard errors are reported.

Figure 5, Figure 6 and Figure 7 represents the data obtained, respectively, for homogeneous, from above, and from below illumination conditions, while in Figure 8 are represented comparatively the data for all the three illumination conditions.

A repeated measured ANOVA revealed a significant main effect for all the 3 manipulated variables, that is, illumination (homogeneous, from above, from below: F (2,28) = 3.3; *p* < 0.05), texture composition (“complete” vs.” incomplete” grid: F (1,14) = 42.8; *p* < 0.001) and texture arrangement (Hughes-type “perspective” grid vs. equidistant “no perspective” grid: F (1,14) = 9.0; *p* < 0.01). Moreover, there were 2 significant interactions, that is, illumination x texture composition (F (2,28) = 3.7; *p* < 0.05), and texture arrangement x texture composition (F (1,14) = 4.9; *p* < 0.05). Bonferroni post-hoc analyses revealed significant differences for the variable illumination for above vs. below conditions only (*p* < 0.05), and for texture arrangement x texture composition interaction for complete (obliques + squares) equidistant “no perspective” grid vs. incomplete (squares only) equidistant “no perspective” grid conditions (*p* < 0.001).

## 4. Discussion 

To summarize, the results highlighted that all three of the manipulated variables led to a significant main effect. As regards the variable illumination condition, the results indicate that the change in the illumination direction has an effect on the illusion and, more specifically, as demonstrated by the post-hoc analyses, that the effect seems to be due to the difference between the illumination from above condition vs. the illumination from below condition. 

With regard to the texture composition variable, the effect, as already noted in the literature, is stronger when the grid is complete, that is, when all types of lines (horizontal, vertical, and oblique) are present. It should be noted that, from the phenomenal point of view, when the obliques are missing, the figural bistability is reduced, that is, the stimulus appears more as a protruding pyramid and less as a corridor. 

As concerns the texture arrangement variable, Hughes-type “perspective” leads to a stronger illusion than equidistant “no perspective” grids. Although from the geometric point of view, both the equidistant and Hughes-type stimuli are incorrect perspectives, it seems that the perceptual system prefers a pseudo-correct perspective compared to the absence of perspective.

The statistically significant effect of the interaction “texture arrangement x texture composition” seems to be mainly determined by the observed difference between the two texture arrangement conditions for the level “absence of obliques” of the variable texture composition. In fact, the illusion is considerably reduced when, in the absence of obliques, we move from a Hughes-type “perspective” stimulus to an equidistant-squares one (that is, absence of perspective).

The effect is stronger when the obliques are present in the stimulus, with no regard to the variable texture arrangement, that is, whether it is a Hughes-type “perspective” or an equidistant “no perspective” grid. The effect, in absolute terms, is weaker when the obliques are missing and the squares are equidistant, even if it must be noted that there is not a significant difference between Hughes-type and equidistant stimuli; this means that, in terms of texture composition, the texture arrangement does not significantly affect the size of the effect. Within the same textural arrangement, for the Hughes-type perspective, the texture composition has no significant effect, that is, there is no difference between the conditions with and without obliques (i.e., complete vs incomplete), while for the equidistant “no perspective” grid conditions the lack of the obliques reduces the illusion.

## 5. Conclusions

In the Art domain, since the Renaissance, the appearance of depth and 3-dimensionality has been elicited by using linear perspective. Relevant experimental literature investigated how linear perspective, geometrical cues, and, more specifically, Hughes’ “reverspectives” like stimuli, act in generating, and which are the determining factors giving rise to the 3D percept [1,2,3,4,12,13,14,15,16,17]. 

Rogers and Gyani [4] demonstrated that a reverspective depicted with a geometrically correct perspective made by pebbles is particularly ineffective, and that one made by a perspective photograph is slightly less effective than a grid. The superior efficacy of Hughes’ reverspectives is further supported by the theory of phenomenal rotation towards the fronto-parallel plane, proposed by Bartel [18,19] discussed in the Introduction.

This fact can lead to the conclusion that the mere presence of oblique lines converging to the vanishing points of reverspectives is as effective as realistically painted textures, only if these textures contain an adequately large number of such oblique lines; if they do not, then the mere presence of oblique lines creates a stronger illusion. Our data for all the three illumination conditions (homogeneous, from above and from below) show that the grid stimulus that contains obliques that are spaced apart in a near-perspective fashion is the most effective among the other experimental stimuli. Two studies that compared “outline” stimuli and their realistically painted versions that contained rich perspective cues are those by Papathomas [2] and by Rogers and Gyani [4].The results of the two studies are somewhat similar regarding the difference between the “outline” stimuli and their realistically painted counterparts. This difference appeared to be more pronounced for Papathomas [2] than for Rogers and Gyani [4], perhaps because of the differences in the stimuli used in the two studies.

From the results, the following conclusions can be drawn: First of all, it is relevant to note that all the textures are effective in evoking the illusion of perspective reversal, although to different extents. In fact, even the least effective stimulus, namely, the grid with only equidistant vertical and horizontal lines, is quite effective, even if it is not a geometrically correct perspective. This result could be considered as a further demonstration of how strong and robust the reverspective illusion can be.

As regards the texture composition, the complete grid stimuli, i.e., the textures that present all components, evoke a more effective illusion than the textures without the oblique elements, regardless of their textural arrangement, that is, Hughes-type “perspective” or equidistantly spaced orthogonal lines (squares) “no perspective”. 

This means that the illusion of perspective reversal is affected by the geometric components of the stimulus’ texture and that these components function optimally only when all types of lines are present. This fact would lead to questioning the importance that Deregowski and Parker [8,9,10] attributed to oblique lines in 3D space perception.

As regards the texture arrangement, the grid stimulus built according to the logic of the artist Patrick Hughes is the most effective one under all the three illumination conditions (i.e., homogeneous, from above and from below), although the one made by equidistant orthogonal lines is also quite effective.

As regards the illumination condition, the influence that the direction of illumination could have on reverspectives perception is an aspect that has never been taken into consideration so far. The present research results seem to suggest that the direction of illumination could affect the strength of the illusion, although, a differential modulation of the effect among the tested illumination directions has not been demonstrated unequivocally. In fact, the lack of neatness of this result is probably due to the main weakness of the present work, that is, the limited number of participants, which could be considered as the main reason for the lack of reliability of the difference found among the different illumination conditions tested. Nonetheless, the most effective illumination direction condition in evoking the effect of perspective reversal seems to be that coming from below, even if it cannot be ruled out that this result could be determined by top-down influences. Under the illumination from below condition, in fact, the stimulus appears as a corridor illuminated from above with the floor appearing brighter than the walls, while the other two illumination directions suggest concavity, not relief. Papathomas observed that a reverspective with a tiled floor elicited a slightly stronger illusion than the same reverspective turned upside-down [3].

In terms of efficacy of the reverspectives, the homogeneous illumination condition seems to be more effective than the illumination from above condition too, even if the difference is not statistically significant. However, it must be noted that even this latter illumination condition (i.e., the light coming from above), has nevertheless elicited the illusion of reversal for all the observers and for all the experimental stimuli. It might be expected that an illumination from above would be less effective than the others since it fully highlights the actual 3D structure of the stimulus; and if we also consider that the visual system assumes that the origin of light is always from above [20], the result that might be expected is that the observer perceives just a pyramid illuminated from above. This, instead, does not occur; indeed, the observer perceives a ceiling of a corridor emanating its own light rather than a protruding pyramid illuminated from above. This fact seems to lead to the conclusion that for the reverspectives, in a conflict between depth cues situation, the stimulus’ texture pictorial cues prevail on other cues such as the motion parallax and the accommodation.

Even if the data we collected are too few to allow one to draw any definitive conclusions, they can however be considered as a starting point for further development of this research. An interesting phenomenon that has been observed that could be further tested in the future, concerns the perceived height of the pyramid. When the switch-off occurs, and the illusory percept of concavity is lost, replaced by the percept of convexity, some observers reported that the height of the illusory recessed pyramid before the switch-off tended to appear larger than that of the actual protruding pyramid, even if this effect quickly disappears. 

Another untested point regards the texture components; that is, the verticals elements were never tested separately from the horizontal ones (i.e., squares were always present). It could be interesting to check their effect individually and/or to misalign them. 

Lastly, since it has been previously indicated [2] that the illusion is affected also by the observer’s movements, that is, if the observer moves along a fronto-parallel plane either laterally or from up and down, the impression of perspective reversal may become more stable and, when the illusion begins to fade, the movement of the observer may renew it. This is a variable that future experiments should be encouraged to take into account to better understand the dynamics of this very unique kind of artwork/visual illusion.
